# Transcriptome analysis of two near-isogenic lines of bell pepper (*Capsicum annuum*) infected with bell pepper endornavirus and pepper mild mottle virus

**DOI:** 10.3389/fgene.2023.1182578

**Published:** 2023-04-13

**Authors:** Cesar Escalante, Noa Sela, Rodrigo A. Valverde

**Affiliations:** ^1^ Department of Entomology and Plant Pathology, Auburn University, Auburn, AL, United States; ^2^ Department of Plant Pathology and Crop Physiology, Louisiana State University, Agricultural Center, Baton Rouge, LA, United States; ^3^ Department of Plant Pathology and Weed Research, The Volcani Center-ARO, Bet-Dagan, Israel

**Keywords:** acute plant viruses, persistent plant viruses, RNA sequencing, tobamovirus, bell pepper endornavirus

## Introduction

Bell peppers (*Capsicum annuum* L) are native plants to the Mesoamerican region, whose domestication likely began in the northeast and east central Mexico (Pickersgill, 1989; Kraft et al., 2013). Among other pepper types, bell peppers are the most commonly cultivated in the United States and worldwide (Pickersgill, 1997; 1989; Eshbaugh, 1993; DeWitt and Bosland, 1996). Plant viruses can be grouped as acute or persistent (Roossinck, 2010). Acute viruses cause symptoms, move from cell-to-cell and systemically, and are transmitted vertically and horizontally. In contrast, persistent plant viruses do not cause symptoms, lack cell-to-cell movement, and are transmitted only vertically *via* gametes. Bell pepper endornavirus (BPEV) is a persistent virus in the family *Endornaviridae* with a genome of approximately 14.7 kb (Okada et al., 2011). BPEV has been reported worldwide, and in the United States, BPEV has been detected in all tested commercial cultivars of bell pepper (Valverde et al., 1990; Okada et al., 2011; Escalante, 2017; Safari and Roossinck, 2018). Like other persistent viruses, BPEV does not cause symptoms in peppers (Fukuhara and Gibbs, 2012). Many acute viruses infect bell pepper, causing severe loses of fruit yield and quality. The acute virus pepper mild mottle virus (PMMoV) is a species of the genus *Tobamovirus* with worldwide distribution, causing economically important diseases of pepper (Pernezny, 2003). This virus is abundant and ubiquitous in nature, and it is transmitted mechanically, through seed, and agricultural tools.

Transcript analysis is one of the most advanced strategies used to determine the flexibility of gene expression as a response to any external or internal stimulus (Dunwell et al., 2001; Mutz et al., 2013). Taking advantage of this technique, identifying genes involved in important traits provides the basis for new progress in the genetic improvement of cultivated plant species (Dunwell et al., 2001). RNA sequencing (RNA-seq) has helped to elucidate different physiological responses of the host plant under infection by plant viruses and abiotic stresses (Li et al., 2016; 2015; Jiao et al., 2020). Using the backcross breeding method, two near-isogenic lines (NILs) of bell pepper cv. Marengo have been developed; one BPEV-infected and the other BPEV-free (Harlan and Pope, 1922; Escalante and Valverde, 2019). The advantage of using these NILs is that we can associate changes in gene expression with the presence of BPEV and not with the genetics of the host. Therefore, we used these NILs of bell pepper to analyze their transcriptome before and after inoculation with PMMoV. The data generated in this study will be useful to further investigate the molecular and biological interactions between persistent and acute viruses in plants.

## Value of the data

Many studies have been conducted evaluating the plant transcriptome response to infection by acute viruses. However, there have been only a few studies on the plant response to persistent viruses. Therefore, using Next-Generation Sequencing (NGS), the transcriptomes of two NILs of bell pepper, before and after infection with the acute virus PMMoV were analyzed. This is the first RNA-seq study reporting the effect of BPEV and PMMoV on the gene expression of the host plant. Therefore, the gene transcript data generated in this study will be useful to further investigate the molecular and biological interactions between endornaviruses and acute plant viruses.

## Materials and methods

### Plant materials

The two NILs of the bell pepper cv. Marengo, one infected with BPEV and the other free of BPEV developed in previous studies, were used in this investigation (Escalante and Valverde, 2019). To assure that acute viruses were not infesting the seed coat, before planting, seeds of the NILs were treated with 10% sodium phosphate tribasic dodecahydrate (Sigma-Aldrich Co., St. Louis, MO) by soaking them in solution for 20 min. Treated seeds were planted in a soil mix consisting of 1.5 parts of soil, 1.5 parts of sand, and 3 parts of potting mix (Miracle-Gro^®^ Lawn Products, Inc., Marysville, OH) in autoclaved clay pots (0.49-L). Plants were grown in the laboratory using artificial light (54W/120V 60Hz/4.0A Lamps) with a photoperiod of 15 h of light and 9 h of dark. Plants were fertilized once with a pelletized fertilizer (Osmocote^®^ Smart-Release^®^ Plant Food 19-6-3, The Scotts Company LLC, Marysville, OH).

### Virus inoculation

Thirty-day-old pepper seedlings were mechanically inoculated with PMMoV. The inoculum consisted of purified PMMoV diluted in phosphate buffer (0.05 M, pH 7.2) to a concentration of 0.05 mg/ml. Three leaves per plant were dusted with silicon carbide (carborundum) and rubbed with inoculum using cotton swabs. The inoculated leaves were rinsed immediately with distilled water. Three plants of each NIL were inoculated with PMMoV, and six plants (three per each NIL) mock-inoculated with phosphate buffer only. Inoculated and mock-inoculated plants were kept in the dark overnight before placing them under fluorescent lights in the laboratory. The inoculation experiments consisted of four treatments: BPEV+/Mock, BPEV-/Mock, BPEV+/PMMoV, and BPEV-/PMMoV (Table 1).

**TABLE 1:** Treatments and summary statistics of RNA-seq data. BPEV = bell pepper endornavirus, and PMMoV = pepper mild mottle virus. The “+” or “-“ symbols indicate infected or free of BPEV, respectively.

Treatment	Accession	Reads (millions)	Bases (Gbp)	Size (G)	GC Content (%)	Average read length
BPEV+/Mock	SRX7131596	32.5	3.2	1.5	44.1	99.26 ± 2.32^a^
BPEV+/Mock	SRX7131587	32.4	3.2	1.5	43.8	99.30 ± 2.17
BPEV+/Mock	SRX7131588	34.0	3.4	1.6	43.6	99.32 ± 2.04
BPEV-/Mock	SRX7131593	35.7	3.5	1.7	44.6	99.22 ± 2.47
BPEV-/Mock	SRX7131594	39.2	3.9	1.8	44.2	99.28 ± 2.21
BPEV-/Mock	SRX7131595	38.7	3.8	1.8	44.4	99.29 ± 2.16
BPEV+/PMMoV	SRX7131590	38.5	3.8	1.4	44.4	98.80 ± 3.75
BPEV+/PMMoV	SRX7131591	39.7	3.9	1.4	44.3	98.77 ± 3.84
BPEV+/PMMoV	SRX7131592	38.6	3.8	1.4	44.9	98.94 ± 3.39
BPEV-/PMMoV	SRX7131585	37.1	3.7	1.3	45.6	98.95 ± 3.38
BPEV-/PMMoV	SRX7131586	42.1	4.2	1.5	43.2	98.87 ± 3.55
BPEV-/PMMoV	SRX7131589	49.9	4.9	1.8	44.4	98.85 ± 3.62

^a^ Represents the standard deviation.

### RNA extraction

Approximately 1.0 g of leaf tissue was collected from each plant 7 days after inoculation. Collected tissue was immediately ground in liquid nitrogen using RNase-free mortar and pestles. Total RNA was extracted from 100 mg of ground tissue using the Plant Total RNA Kit (Spectrum™, Sigma-Aldrich Co., St. Louis, MO). Total RNA was treated with DNase for 30 min using the On-Column DNase I Digestion Kit (Sigma-Aldrich Co.). Total RNA concentration and quality control were determined using a nanodrop ND-1000 spectrophotometer (NanoDrop^®^ Technologies, Inc., Wilmington, DE). RNA integrity was evaluated by electrophoresis in 1% agarose gel. The total RNA was stabilized to be handled at room temperature using the RNA Stable Tube Kit (Sigma-Aldrich Co.). Liquid-nitrogen ground tissue was used to extract dsRNA to confirm the presence or absence of BPEV in the two NILs using the method described by Khankhum et al. (2017). Reverse transcription polymerase chain reaction (RT-PCR) was also used to test viral infections (Jarret et al., 2008; Okada et al., 2011).

### Library construction and RNA-seq

The RNA was used for Illumina mRNA NGS. Ribosomal RNA (rRNA) was depleted using the RiboZero kit (Epicentre^®^, Madison, WI). The rRNA-depleted total RNA was converted to first-strand cDNA using the SuperScript™ kit (Invitrogen™, Waltham, MA) random primers, and ds-cDNA was generated after RNA hydrolysis. A total of 12 RNA-seq libraries were prepared with the TruSeq Stranded mRNAseq Sample Prep Kit (Illumina^®^ Inc., Hayward, CA). Paired-end reads for Illumina mRNA sequencing of 100 nt in length were generated prior to bar-coding, and subjected to the appropriate protocols. The libraries were pooled in equimolar concentration, quantified by quantitative PCR, and sequenced on one lane for 101 cycles from one end of the fragments on a HiSeq2500 using a HiSeq SBS sequencing kit version 4.0. Fastq files were generated and multiplexed with the bcl2fastq v2.17.1.14 Conversion Software (Illumina Inc.).

### Transcriptome assembly and data analysis

**FIGURE 1:**
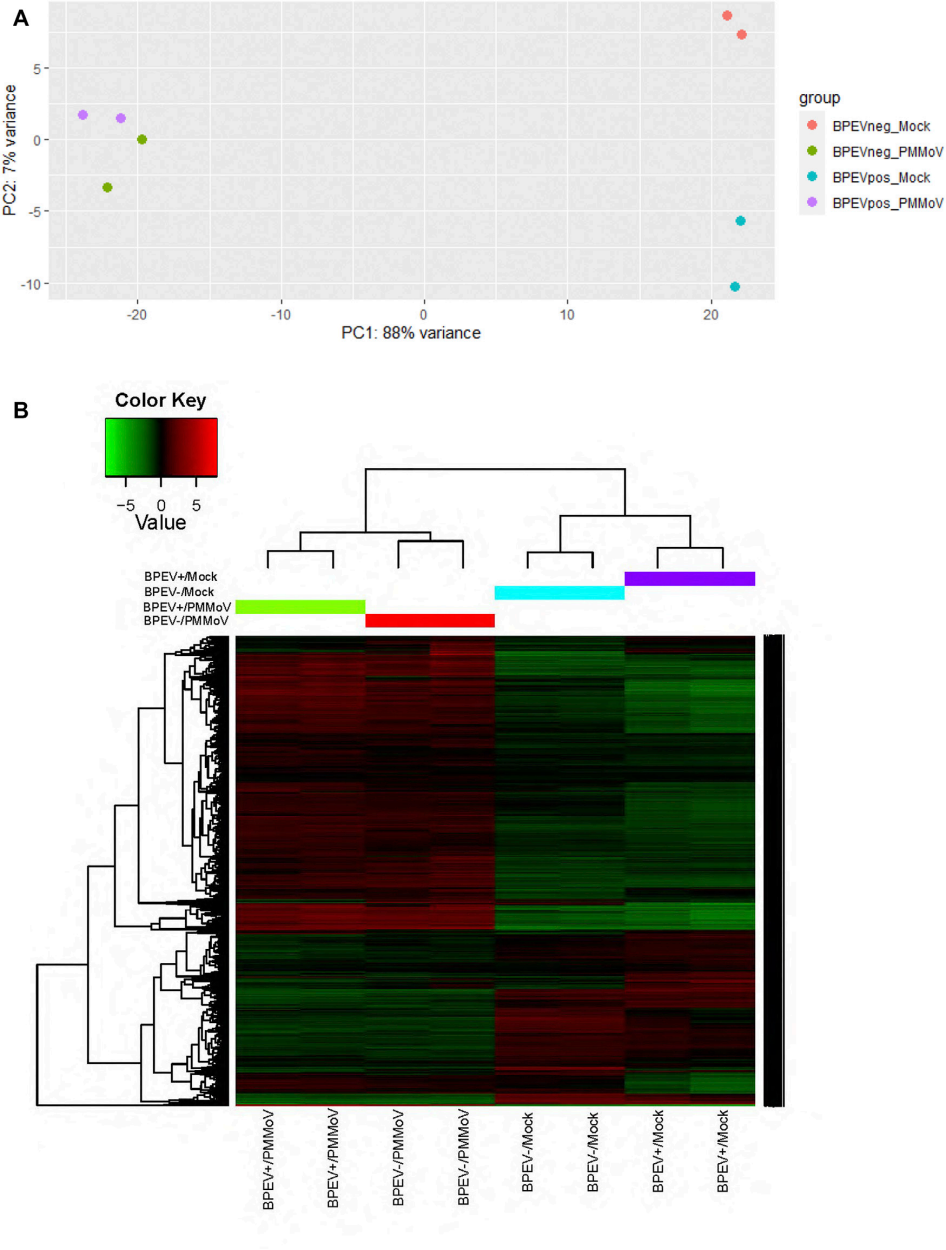
**(A)** Principal component analysis **(B)** Heat map of the differentially expressed genes of selected libraries from the RNA-seq of two bell pepper near-isogenic lines. Red and green indicate up- and downregulated genes, respectively (*p* < 0.1). BPEV+ = infected with bell pepper endornavirus, BPEV- = free of bell pepper endornavirus, and PMMoV = pepper mild mottle virus.

The RNA transcriptome assembly was conducted by mapping the 12 libraries to the pepper reference genome version 1.55 in the Solanaceae Genomics Network (https://solgenomics.net/). Before transcriptome assembly, adaptors were trimmed from the reads. Transcriptome assembly was performed using Bowtie version 1.2.2 (Langmead and Salzberg, 2012). Before conducting the Differentially Expressed Genes (DEG) analysis, a principal component analysis test was performed to determine if the 12 libraries were uniform (Figure 1A). After the test, some libraries did not correlate. Therefore, to maintain uniformity in the number of biological repetitions, one library was removed from each treatment to perform the DEG analysis. Using the RNA-seq data, the total number of differentially expressed genes (*p* < 0.1 and *p* < 0.05) were identified using the package DESeq2 version 1.30.1 in R software (Love et al., 2014). The main biological processes, molecular functions, and cellular components of the DEGs were determined by performing Gene ontology (GO) analysis using the Blast2GO methodology in OmicsBox version 1.4.11 (Götz et al., 2008) and the Gene Ontology Resource web page (http://geneontology.org/).

## Results

A total of 458.3 million reads were obtained from the 12 libraries. This corresponds to 245.8 million reads from the six libraries infected with PMMoV and 212.5 million reads from the mock-inoculated libraries (Table 1). Three conditions were generated to analyze DEGs: mock-inoculated plants infected with BPEV vs mock-inoculated plants free of BPEV (BPEV+/Mock vs BPEV-/Mock), plants double infected with BPEV and PMMoV vs mock-inoculated plants free of BPEV (BPEV+/PMMoV vs BPEV-/Mock), and plants infected only with PMMoV vs mock-inoculated plants free of BPEV (BPEV-/PMMoV vs BPEV-/Mock). A description of the 100 highly DEGs (based on the adjusted *p*-value) is presented in Supplementary Tables S2-S4. When compared with PMMoV-infected plants, a lower number of genes were differentially expressed in both NILs free of this virus (Figure 1B and Supplementary Table S1). BPEV-infected plants inoculated with PMMoV showed a six-fold increase of upregulated genes when compared to BPEV-infected plants that were mock-inoculated. Most of the upregulated genes in PMMoV-infected plants (in both NILs) are associated with responses to biotic and abiotic stimuli (Supplementary Figure S1). The expression of lesser genes in BPEV-infected mock-inoculated plants suggests that BPEV does not represent a major disruption of normal gene expression and metabolism for the host. In contrast, PMMoV has been reported to be an aggressive pathogen that can trigger a higher number of genes in the pepper host (Jiao et al., 2020). In transcriptomics studies of PMMoV-infected plants, the main biological processes regulated after infection were the response to biotic stimulus (Jiao et al., 2020). In this investigation, we obtained similar results when pepper plants were inoculated with PMMoV in single or mixed infection with BPEV. When plants were only infected with BPEV, some genes involved in response to the biotic stress were upregulated. Interestingly, similar results were observed in other studies when pepper plants were exposed to heat or chilling stresses (Li et al., 2015; Li et al., 2016).

The expression of genes that have been previously reported to respond to biotic and abiotic stimuli indicates that BPEV has an active interaction with the host, which could be parasitic, although to a lesser extent when compared to the effect of acute viruses. This interaction is reflected by the significant number of upregulated genes which included lipid transfer protein LTP1 precursor and PR-10 type pathogenesis-related protein. These genes have been reported to be involved in the plant defense response. This is not surprising as it has been reported that BPEV can cause cellular ultrastructural changes in bell pepper (Otulak-Koziel et al., 2020). Transcriptomic analysis of two lines (not NILs) of common bean, one infected with Phaseolus vulgaris endornavirus and the other endornavirus-free, revealed several upregulated genes involved in response to pathogen infection and environmental stresses (Khankhum et al., 2016). Furthermore, oxidation-reduction processes were found to be associated with endornavirus infection. Although the common bean lines were not NILs, the biological processes associated with endornavirus infection in common bean were similar to the results obtained in our investigation.

In pepper, several examples illustrate beneficial host-pathogen interactions (Kim et al., 2021; Liu et al., 2021; Zhang et al., 2021). For example, *Paenibacillus polymyxa*, a bacterium associated with promoting stem and leaf growth on pepper, enhanced the host immune system and induced systemic resistance to pathogens (Liu et al., 2021). Determining the different mechanisms involved in response to pathogen infection could provide essential information to further study the interactions among BPEV, the host, and acute viruses. The gene transcript information provided in this investigation will be useful to further study these mechanisms. Environmental factors have been associated with variations in plant fitness and resistance to pathogens in pepper as a response to gene regulation after exposing the pepper plants to these abiotic conditions (Kim et al., 2021; Zhang et al., 2021). Coupling the infection of BPEV with temperature fluctuations and CO_2_ concentrations can provide insights into the activation of pathogen resistance genes in pepper.

## Data Availability

The datasets presented in this study can be found in online repositories. The names of the repository/repositories and accession number(s) can be found at: https://www.ncbi.nlm.nih.gov/, PRJNA588750.
