# Supplementary material for: Transcriptome analysis of two near-isogenic lines of bell pepper (Capsicum annuum) infected with bell pepper endornavirus and pepper mild mottle virus
Source: Front Genet. 2023 Apr 13;14:1182578. doi: 10.3389/fgene.2023.1182578 (PMC10133535; doi:10.3389/fgene.2023.1182578)
Supplement: Supplementary file 1 [file DataSheet1.zip › Supplementary_Figure_1.pdf]

Supplementary Material

A

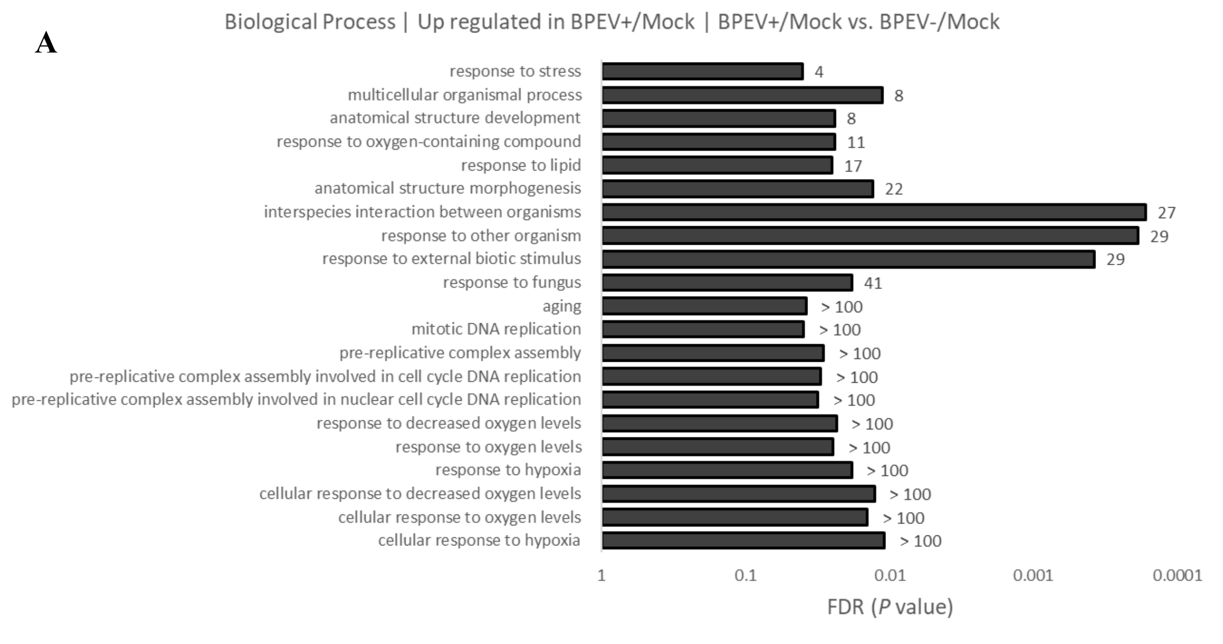

Figure S1. Gene ontology analysis of differentially expressed genes of two bell pepper near-isogenic lines. (A-E) Biological process, molecular function and cellular component up- and downregulated in BPEV+/Mock for condition BPEV+/Mock vs. BPEV-/Mock. (F-K) Biological process, molecular function and cellular component up- and downregulated in BPEV+/PMMoV for condition BPEV+/PMMoV vs. BPEV-/Mock. (L-Q) Biological process, molecular function and cellular component up- and downregulated in BPEV-/PMMoV for condition BPEV-/PMMoV vs. BPEV-/Mock. Biological processes are represented with black bars, molecular functions are represented with white bars and cellular components are presented with gray bars. Conditions are described at the top of each graphic. BPEV+ = infected with bell pepper endornavirus, BPEV- = free of bell pepper endornavirus, PMMoV = pepper mild mottle virus, and FDR = false discovery rate. Values at the bar end indicate enrichment fold change. No significant upregulated molecular functions were observed for condition BPEV+/Mock vs. BPEV-/Mock. (figure continued).

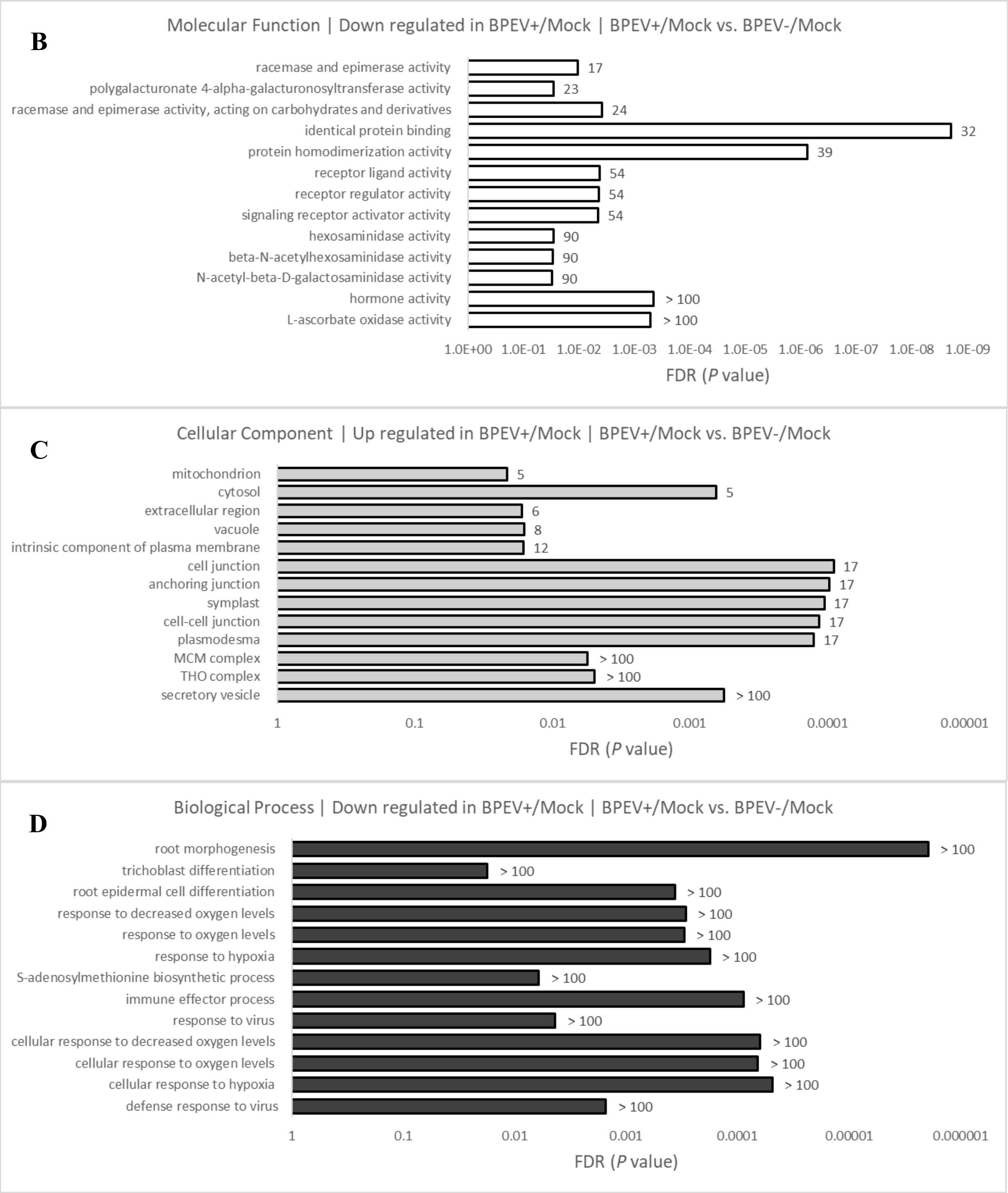

Figure S1. Continued.

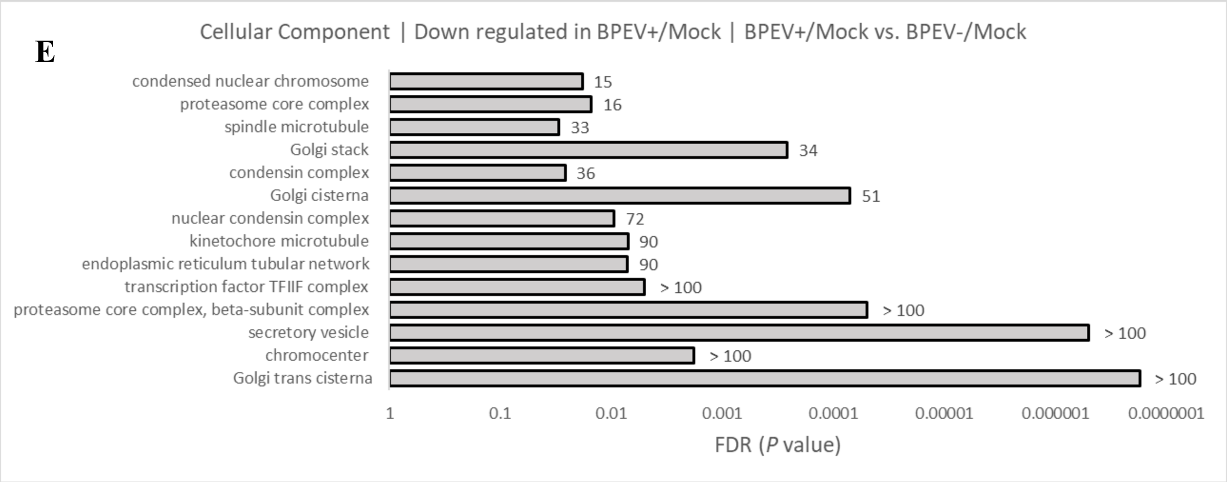

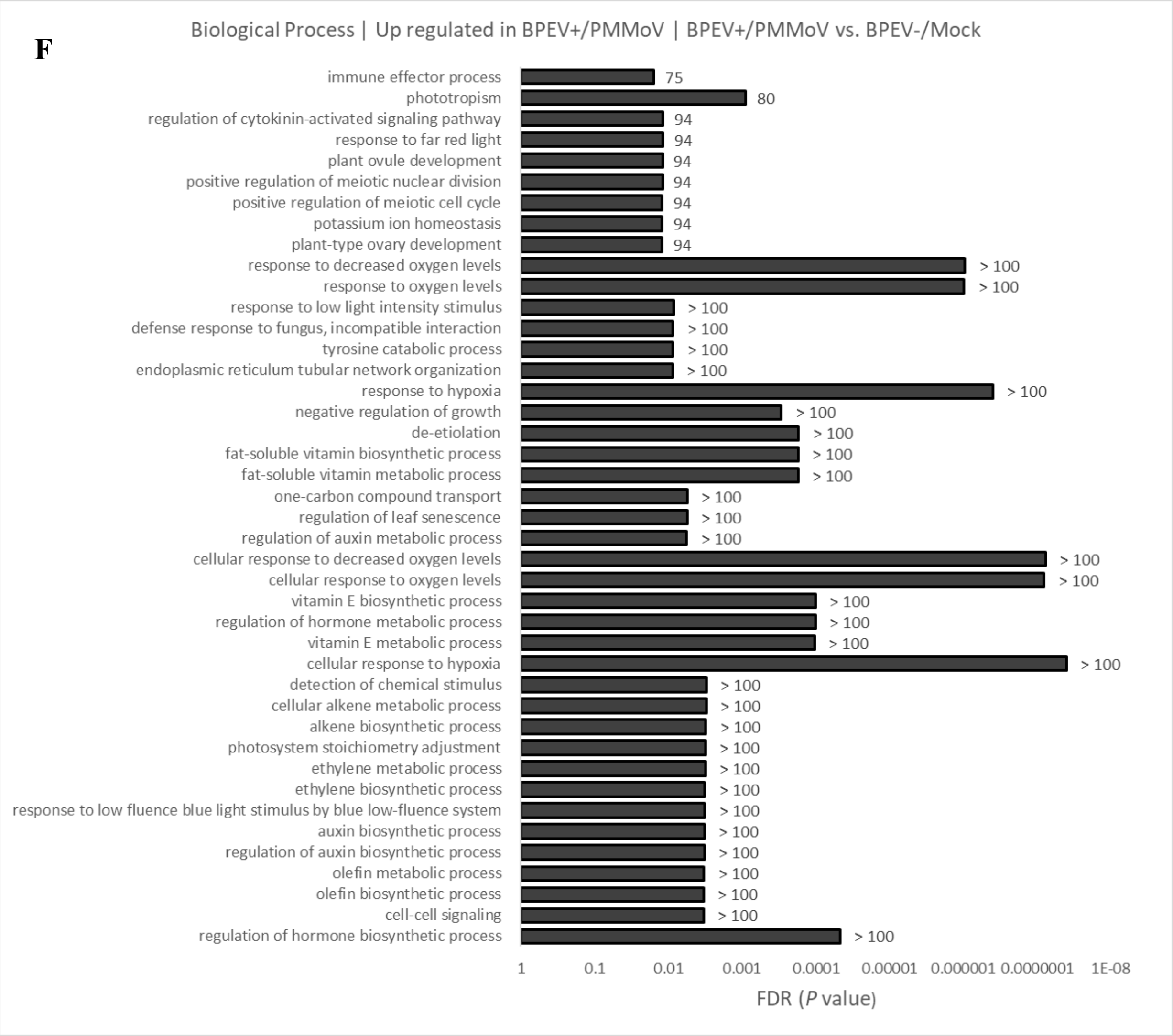

Figure S1. Continued.

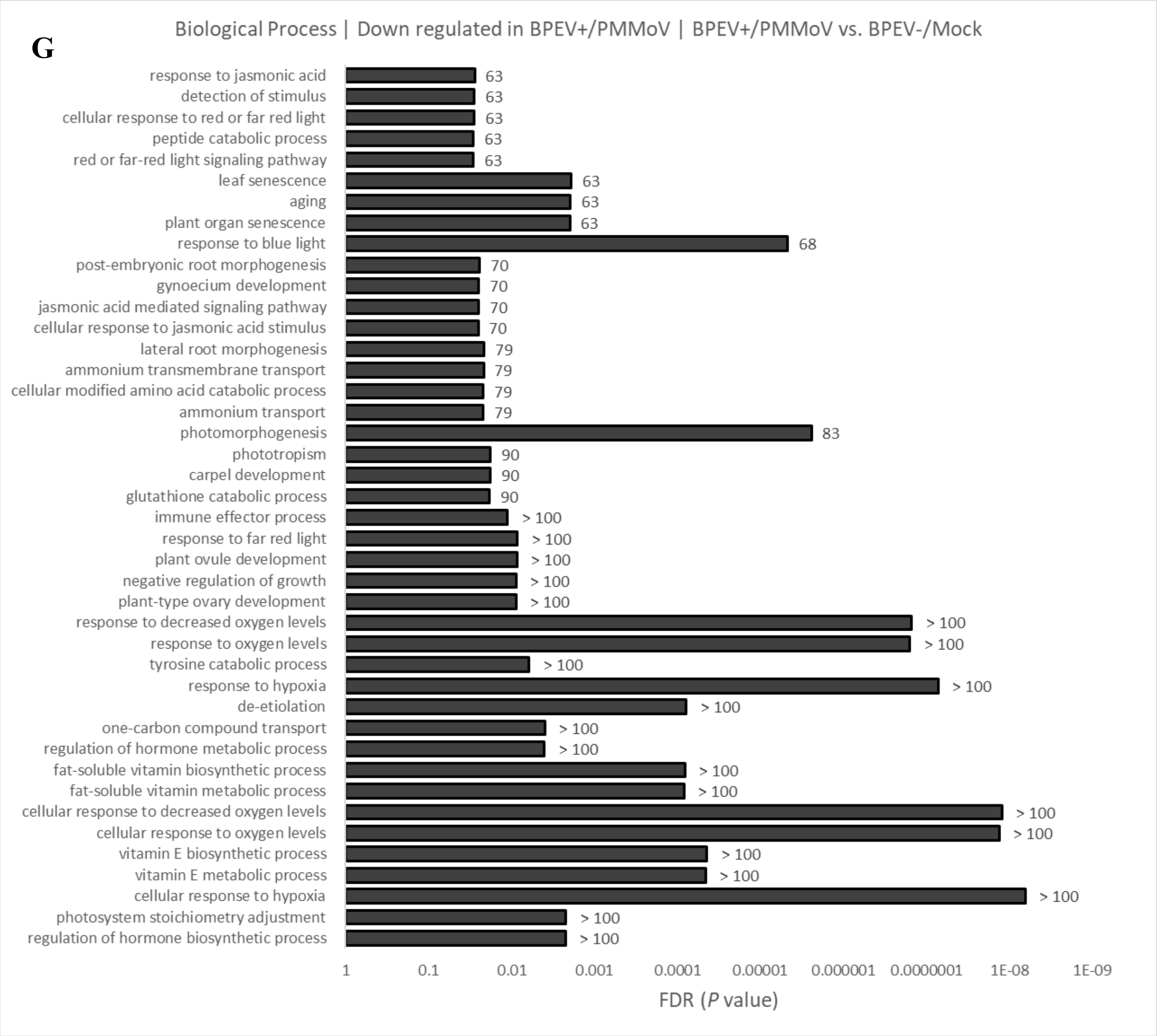

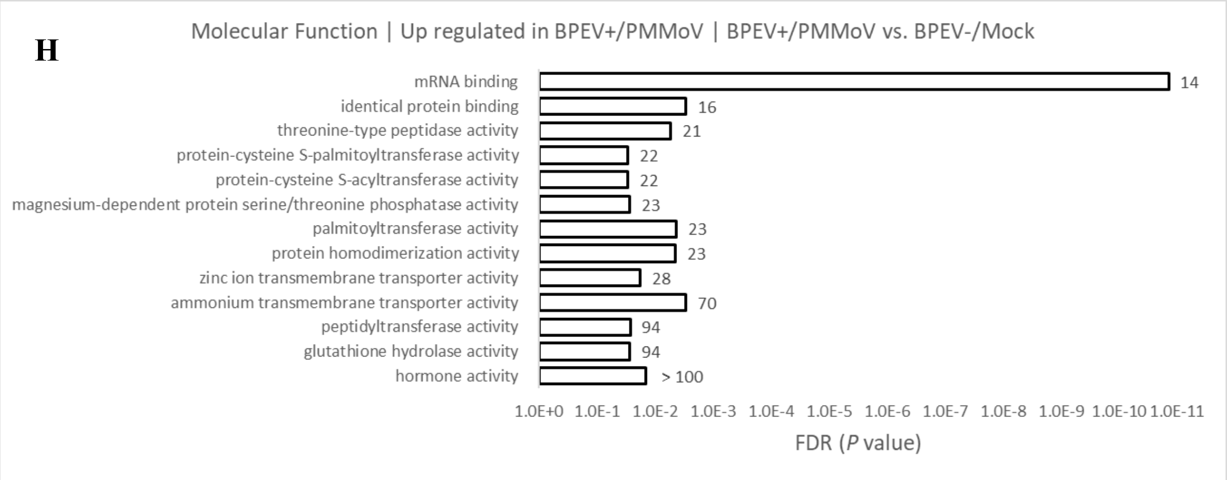

Figure S1. Continued.

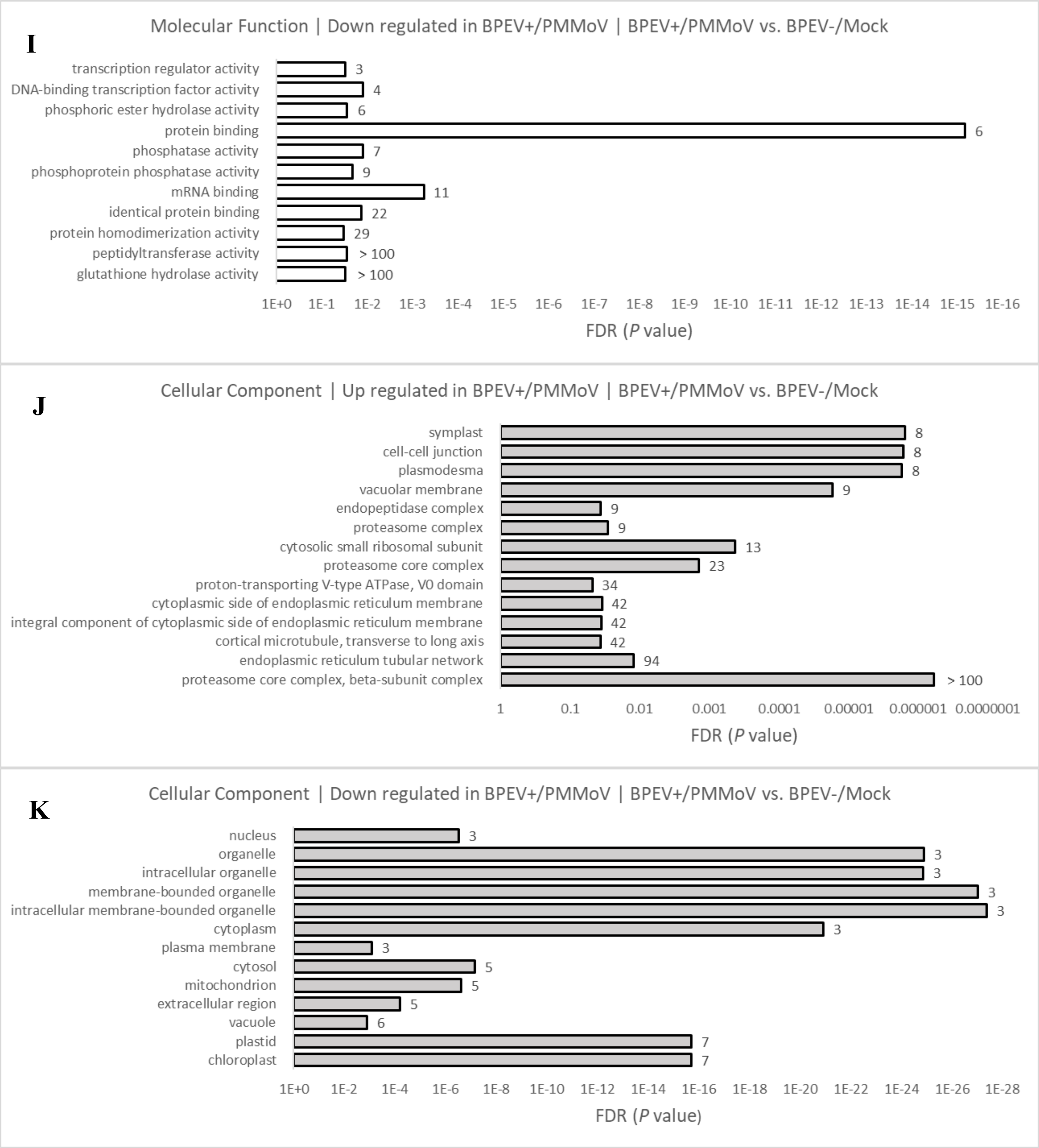

Figure S1. Continued.

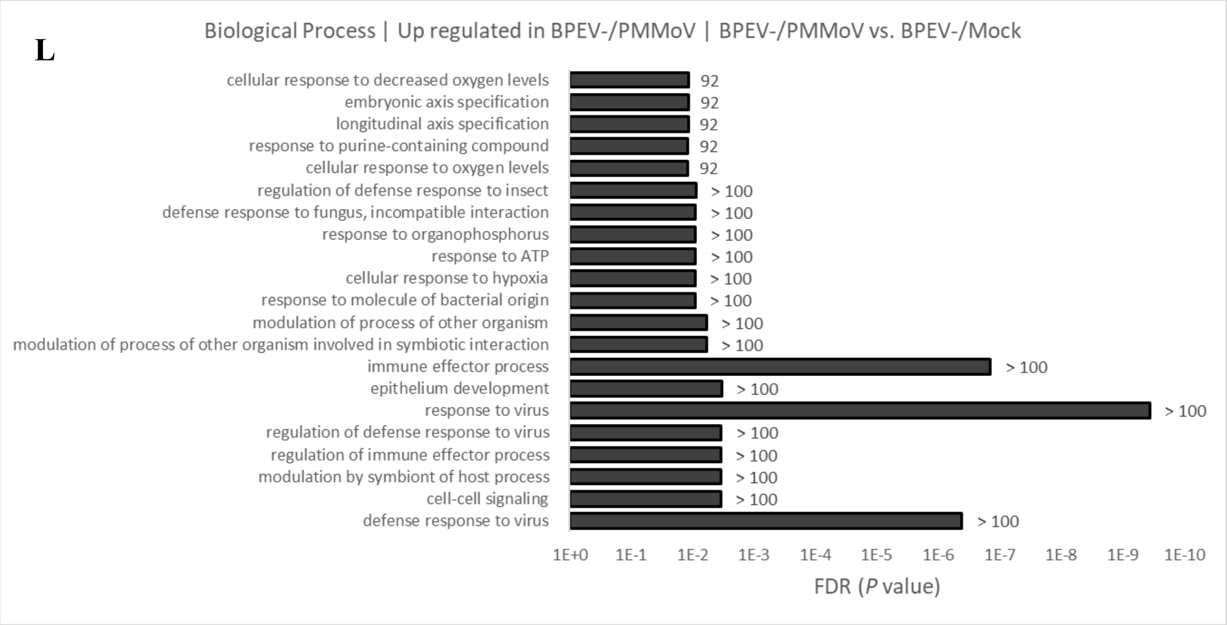

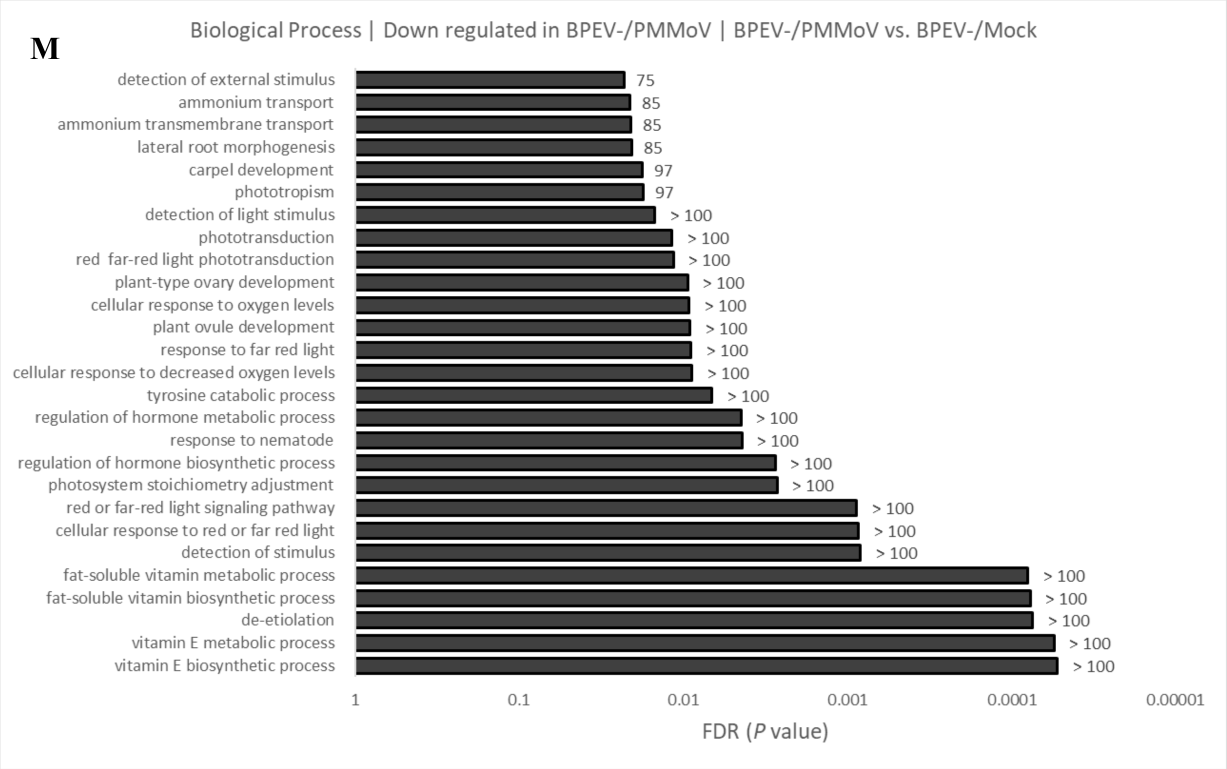

Figure S1. Continued.

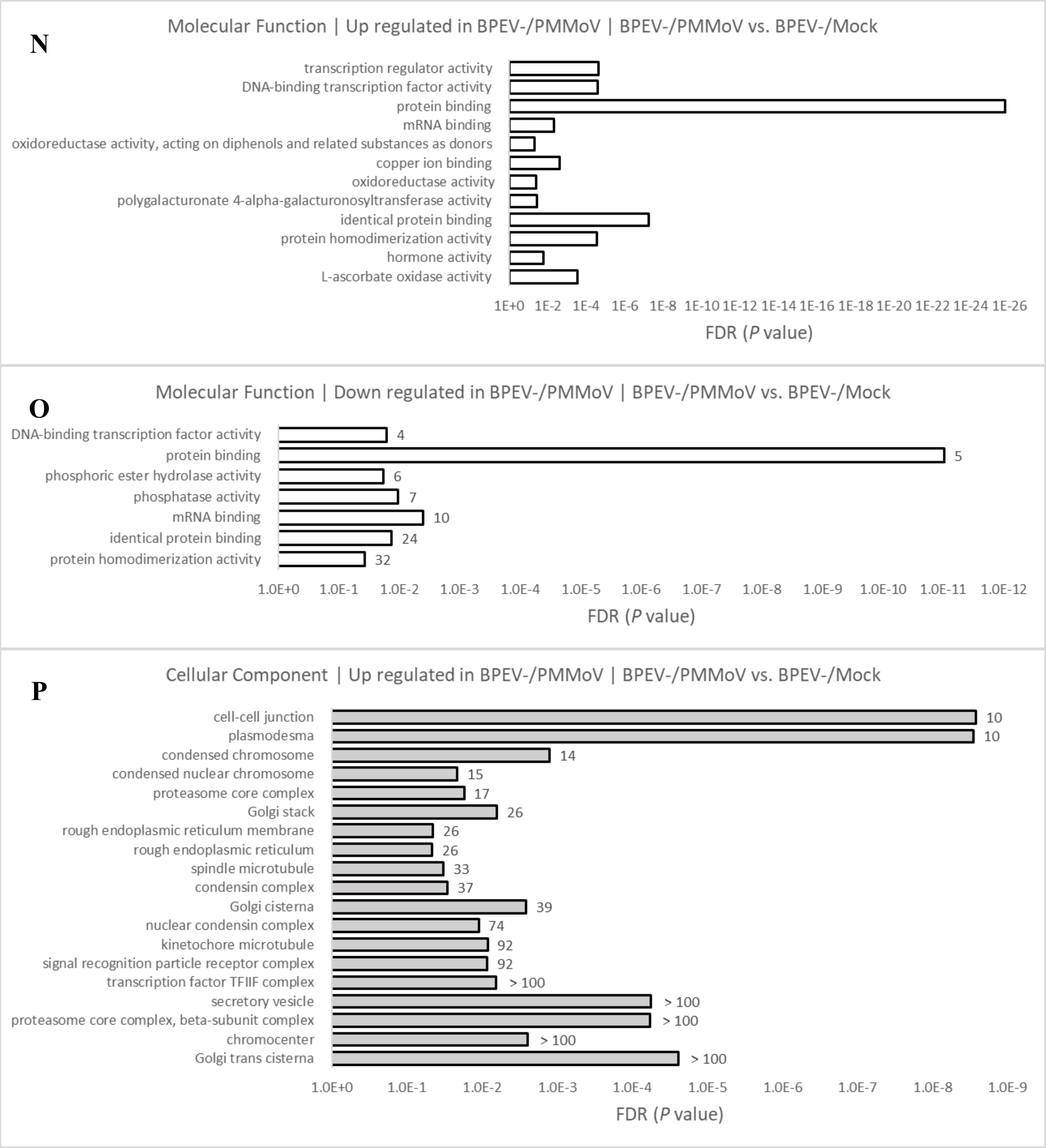

Figure S1. Continued.

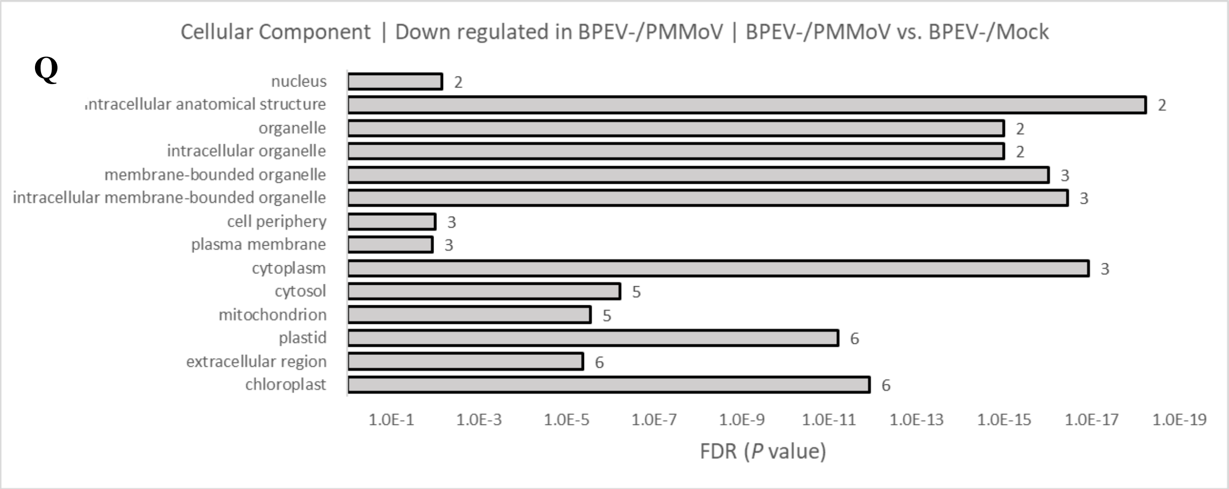

Figure S1. Continued.
