# Supplementary material for: Transcriptome analysis of two near-isogenic lines of bell pepper (Capsicum annuum) infected with bell pepper endornavirus and pepper mild mottle virus
Source: Front Genet. 2023 Apr 13;14:1182578. doi: 10.3389/fgene.2023.1182578 (PMC10133535; doi:10.3389/fgene.2023.1182578)
Supplement: Supplementary file 1 [file DataSheet1.zip › Supplementary_Table_1.pdf]

Supplementary Material

Table S1. Summary statistics of differentially expressed genes. BPEV+ = bell pepper endornavirus positive, BPEV- = bell pepper endornavirus negative, and PMMoV = pepper mild mottle virus, ND = not determined.

**[1]BPEV+/Mock vs. BPEV-/Mock**		
	**Genes (p < 0.1)**	**Genes (p < 0.05)**
Up-regulated	342.0	275.0
Down-regulated	852.0	674.0
Outliers	0.0	ND
Low counts	11158.0	ND
**[2]BPEV+/PMMoV vs. BPEV-/Mock**		
Up-regulated	2,083.0	1,722.0
Down-regulated	1,683.0	1,425.0
Outliers	0.0	ND
Low counts	6,347.0	ND
**[3]BPEV-/PMMoV vs. BPEV-/Mock**		
Up-regulated	2,016.0	1,696.0
Down-regulated	1,615.0	1,397.0
Outliers	0.0	ND
Low counts	6437.0	ND

[1] Regulated in BPEV+/Mock samples.
[2] Regulated in BPEV+/PMMoV samples.
[3] Regulated in BPEV-/PMMoV samples.
