# Supplementary material for: Transcriptome analysis of two near-isogenic lines of bell pepper (Capsicum annuum) infected with bell pepper endornavirus and pepper mild mottle virus
Source: Front Genet. 2023 Apr 13;14:1182578. doi: 10.3389/fgene.2023.1182578 (PMC10133535; doi:10.3389/fgene.2023.1182578)
Supplement: Supplementary file 1 [file DataSheet1.zip › Supplementary_Table_2.pdf]

Supplementary Material

Table S2. One hundred highly differentially expressed genes that resulted from the transcriptome analysis of two bell pepper near-isogenic lines in BPEV+/Mock for condition BPEV+/Mock vs. BPEV-/Mock. Genes were selected based on the adjusted p value. BPEV+ = infected with bell pepper endornavirus, BPEV- = free of bell pepper endornavirus, and ND = not determined. (Table continued).

Pepper ID	Gene Description	Log2 Fold Change	LFC SE	*P* adj
CA10g08490	Lipid transfer protein LTP1 precursor	3.9	0.4	3.90E-21
CA12g16570	Cysteine protease	2.4	0.3	6.20E-16
CA01g24020	Stem-specific protein TSJT1	1.9	0.2	3.50E-14
CA07g20630	Heat shock cognate protein 80	1.8	0.2	1.10E-13
CA10g19080	Uncharacterized protein LOC101267365	2.5	0.3	1.60E-11
CA06g02390	GILT-like protein	3.7	0.5	2.80E-11
CA12g16580	Cysteine protease	2.4	0.3	1.30E-10
CA00g45250	PR-10 type pathogenesis-related protein	2	0.3	1.30E-10
CA03g15850	Pectin methlyesterase inhibitor protein 1	3.6	0.5	5.10E-10
CA08g17890	Uncharacterized protein LOC102591329	2.4	0.3	6.20E-10
CA00g89810	H1 histone-like protein	1.5	0.2	1.90E-09
CA11g17720	Histone deacetylase HDT2-like	1.6	0.2	2.70E-09
CA12g07660	DNAJ protein homolog isoform X1	1.3	0.2	4.40E-09
CA08g01260	Probable ascorbate-specific transmembrane electron transporter 1	3.5	0.5	5.40E-09
CA12g14030	Actin-104	1.7	0.3	4.00E-08
CA06g19870	BTB POZ and TAZ domain-containing protein 1-like	2.4	0.4	7.20E-08
CA00g72880	Caffeoylshikimate esterase-like	4.6	0.7	1.10E-07
CA12g22410	Zinc finger A20 and AN1 domain-containing stress-associated protein 5-like	1.3	0.2	1.10E-07
CA04g18690	Uncharacterized protein LOC102596791 isoform X1	3.4	0.6	1.10E-07
CA02g03340	Uncharacterized protein LOC104228512	2.6	0.4	1.50E-07
CA02g15240	Auxin-repressed protein ARP1	1.5	0.2	2.60E-07
CA01g18060	Uncharacterized protein LOC104245894 isoform X2	1.5	0.2	2.90E-07
CA03g30440	Uncharacterized protein LOC102591281	3.6	0.6	3.70E-07
CA01g19950	Zinc finger A20 and AN1 domain-containing stress-associated protein 8	1.3	0.2	4.30E-07

Pepper ID	Gene Description	Log2 Fold Change	LFC SE	*P* adj
CA08g17910	Uncharacterized protein LOC102591329	2.3	0.4	5.70E-07
CA06g23330	Uncharacterized RNA-binding protein	1.4	0.2	9.30E-07
CA00g09910	Cytosolic NADP-malic enzyme	2.2	0.4	1.00E-06
CA06g05410	Phosphomethylpyrimidine chloroplastic	1.7	0.3	1.40E-06
CA03g25730	High mobility group nucleosome-binding domain-containing protein 5-like	1.6	0.3	2.10E-06
CA12g17600	Bifunctional nuclease 1-like	1.6	0.3	2.20E-06
CA12g20940	UPF0396 protein CG6066	2.3	0.4	3.20E-06
CA10g21470	Probable ribosome biogenesis protein RLP24	1.1	0.2	3.20E-06
CA12g09270	Magnesium-dependent phosphatase 1-like	1.7	0.3	3.60E-06
CA12g03320	Homeobox-leucine zipper protein ATHB-6-like	1.4	0.2	4.00E-06
CA11g19970	Glutaredoxin-C6-like	1.7	0.3	4.30E-06
CA09g13890	Heterogeneous nuclear ribonucleoprotein 1-like	1.1	0.2	5.00E-06
CA12g22670	Protein eceriferum 1-like	4.2	0.8	6.60E-06
CA12g03310	Homeobox-leucine zipper protein ATHB-6-like	1.7	0.3	7.00E-06
CA07g11190	ACC oxidase	2.2	0.4	7.20E-06
CA10g21700	Uncharacterized protein LOC101268236	2	0.4	8.40E-06
CA09g04850	Heat shock cognate 70 kDa protein 2-like	1.2	0.2	8.50E-06
CA08g18480	Protein early flowering 4-like	1.9	0.4	1.10E-05
CA04g20830	Uncharacterized protein LOC104105807	1.9	0.4	1.50E-05
CA07g19520	Thiamine thiazole chloroplastic	0.9	0.2	1.50E-05
CA01g30410	Cation transport regulator-like protein 2-like	1	0.2	2.80E-05
CA02g12750	Subtilisin-like protease	2.3	0.5	2.80E-05
CA06g06590	ND	0.9	0.2	3.00E-05
CA04g22720	ND	1.6	0.3	3.00E-05
CA01g10370	Probable carboxylesterase 8	1.1	0.2	3.20E-05
CA02g26580	Proline oxidase dehydrogenase 1	2	0.4	3.50E-05
CA00g95270	Stress-induced protein partial	-5.5	0.4	7.30E-44
CA07g01490	Stress-induced protein partial	-5.5	0.4	7.30E-44
CA10g02470	Thionin-like protein	-6.1	0.5	3.80E-36
CA10g02530	Thionin-like protein	-2.7	0.2	1.20E-23
CA10g18950	Miraculin-like	-2.5	0.2	9.70E-23

Pepper ID	Gene Description	Log2 Fold Change	LFC SE	*P* adj
CA05g02660	BURP domain-containing protein 3-like	-2.5	0.3	1.10E-18
CA07g03730	ND	-2	0.2	1.20E-17
CA02g09720	KUNITZ-type protease inhibitor precursor	-4.4	0.5	7.00E-17
CA07g13950	Cell wall protein	-4.3	0.5	1.80E-15
CA05g11420	Uncharacterized protein LOC102601302	-2.6	0.3	1.10E-13
CA07g21150	Phosphoglycerate chloroplastic	-1.7	0.2	3.00E-13
CA02g05510	Stress-induced protein 16	-1.8	0.2	3.10E-13
CA06g23480	Histone H2B-2	-2.1	0.3	2.40E-12
CA10g16990	Thioredoxin partial	-2	0.3	2.60E-12
CA08g14370	Chloroplast ferredoxin	-1.6	0.2	9.30E-12
CA00g15320	Linoleate 13S-lipoxygenase 2- chloroplastic-like	-1.7	0.2	3.00E-11
CA01g01250	Uncharacterized protein LOC104219001	-3.3	0.4	5.60E-11
CA03g30170	Chitin-binding lectin 1-like	-4.1	0.5	5.60E-11
CA03g36980	Pectinesterase inhibitor U1	-1.8	0.2	6.80E-11
CA02g26820	Protein GAST1-like	-2.1	0.3	8.30E-11
CA08g08870	Protein aspartic protease in guard cell 2-like	-5.3	0.7	1.10E-10
CA05g05760	Glyceraldehyde-3-phosphate dehydrogenase chloroplastic	-1.5	0.2	1.40E-10
CA07g00200	Hypersensitive response assisting protein	-2.2	0.3	1.70E-10
CA05g20680	Chlorophyll a-b binding protein chloroplastic-like	-23.5	3.2	1.80E-10
CA07g11720	Glycine-rich cell wall structural protein 1-like	-5.2	0.7	2.50E-10
CA03g34630	Aquaporin TIP2-1-like	-3.1	0.4	3.00E-10
CA02g07770	Oxygen-evolving enhancer protein chloroplastic	-1.3	0.2	4.70E-10
CA01g24340	Glutamine chloroplastic	-1.4	0.2	1.00E-09
CA07g04920	Heat shock protein 83	-2	0.3	1.20E-09
CA02g29600	Carbonic anhydrase	-2.2	0.3	1.30E-09
CA10g22220	Photosystem II core complex proteins chloroplastic	-1.6	0.2	1.50E-09
CA07g04930	ND	-2.5	0.4	2.60E-09
CA09g16980	Uncharacterized protein LOC102595674	-2.2	0.3	3.20E-09
CA06g23490	Histone H2B-2	-1.4	0.2	4.10E-09
CA05g00450	Uncharacterized protein LOC101243677	-1.9	0.3	7.50E-09
CA01g19880	Thylakoid lumenal 19 kDa chloroplastic	-1.8	0.3	1.00E-08

Pepper ID	Gene Description	Log2 Fold Change	LFC SE	*P* adj
CA09g01970	S-adenosylmethionine synthase 3	-1.2	0.2	1.10E-08
CA08g17980	Uncharacterized protein LOC101256427	-1.5	0.2	1.10E-08
CA11g17600	Uncharacterized protein At5g22580-like	-2	0.3	1.10E-08
CA09g10320	Chlorophyll a-b binding protein chloroplastic-like	-1.5	0.2	1.20E-08
CA05g03420	Histone H4	-2	0.3	1.20E-08
CA10g22340	Magnesium-protoporphyrin IX monomethyl ester	-1.5	0.2	1.60E-08
CA01g21950	Histone -like	-2.4	0.4	1.80E-08
CA07g03740	Defensin-like protein	-2.3	0.4	1.80E-08
CA06g13900	14 kDa proline-rich	-2.9	0.5	5.50E-08
CA06g21850	Chloroplast stem-loop binding protein of 41 kDa chloroplastic	-1.5	0.2	6.40E-08
CA03g29990	Geranylgeranyl reductase	-2	0.3	9.40E-08
CA06g11950	Chloroplast chlorophyll a b-binding protein	-1.1	0.2	1.00E-07
CA02g15060	Pistil-specific extensin-like protein	-3.3	0.5	1.10E-07
CA03g31890	Palmitoyl-monogalactosyldiacylglycerol delta-7 chloroplastic	-2.1	0.3	1.10E-07
