# Supplementary material for: Transcriptome analysis of two near-isogenic lines of bell pepper (Capsicum annuum) infected with bell pepper endornavirus and pepper mild mottle virus
Source: Front Genet. 2023 Apr 13;14:1182578. doi: 10.3389/fgene.2023.1182578 (PMC10133535; doi:10.3389/fgene.2023.1182578)
Supplement: Supplementary file 1 [file DataSheet1.zip › Supplementary_Table_3.pdf]

Supplementary Material

Table S3. One hundred highly differentially expressed genes that resulted from the transcriptome analysis of two bell pepper near-isogenic lines in BPEV+/PMMoV for condition BPEV+/PMMoV vs. BPEV-/Mock. Genes were selected based on the adjusted p value. BPEV+ = infected with bell pepper endornavirus, BPEV- = free of bell pepper endornavirus, PMMoV = pepper mild mottle virus, and ND = not determined. (Table continued).

Pepper ID	Gene Description	Log2 Fold Change	LFC SE	*P* adj.
CA00g61790	Protein ELF4-like 3-like	5.1	0.2	4.80E-102
CA04g18300	WRKY transcription factor 2	6.7	0.3	3.90E-101
CA03g06510	GDP-mannose 3-epimerase	3.5	0.2	5.10E-83
CA08g13400	Uncharacterized protein LOC102600962	6.4	0.3	1.50E-75
CA00g83460	rRNA intron-encoded homing endonuclease	6.3	0.3	4.90E-71
CA00g91060	rRNA intron-encoded homing endonuclease	6.3	0.3	4.90E-71
CA00g81400	rRNA intron-encoded homing endonuclease	6.3	0.3	4.90E-71
CA00g93960	rRNA intron-encoded homing endonuclease	6.3	0.3	4.90E-71
CA00g91600	rRNA intron-encoded homing endonuclease	6.3	0.3	4.90E-71
CA12g18930	Ankyrin repeat and protein kinase domain-containing protein 1-like	5.3	0.3	5.30E-71
CA00g83820	Tubulin alpha-3 chain	3.6	0.2	8.90E-68
CA03g23600	Hypothetical protein (mitochondrion)	5.9	0.4	4.40E-56
CA06g22700	CCR4 associated factor 1-related protein	4.2	0.3	1.30E-53
CA00g67540	Vesicle-associated protein 1-3-like	5	0.3	7.30E-51
CA10g17080	Protein YLS9-like	3.3	0.2	6.60E-50
CA06g07430	60s ribosomal protein l36-2-like	2.6	0.2	1.50E-47
CA06g26670	60s ribosomal protein l27-like	2.7	0.2	9.00E-44
CA12g07360	Pentatricopeptide repeat-containing protein chloroplastic-like	3.8	0.3	1.60E-42
CA00g95020	Hypothetical protein MTR_5g051150	4.4	0.3	1.90E-42
CA00g94500	Hypothetical protein MTR_5g051150	4.4	0.3	1.90E-42
CA03g20640	Hypothetical protein CICLE_v10027385mg, partial	4.6	0.3	3.10E-41
CA01g02010	PIN2 TERF1-interacting telomerase inhibitor 1 isoform X1	3.2	0.2	3.30E-40
CA01g22170	40s ribosomal protein S16-like	2.2	0.2	3.40E-39

Pepper ID	Gene Description	Log2 Fold Change	LFC SE	*P* adj.
CA10g01770	Protein transport protein SEC61 subunit alpha-like	3.1	0.2	8.00E-38
CA03g36540	Probable xyloglucan endotransglucosylase hydrolase	3.7	0.3	1.80E-36
CA00g91590	Hypothetical protein SORBIDRAFT_1138s002030	3.9	0.3	3.10E-36
CA00g83490	Hypothetical protein SORBIDRAFT_1138s002030	3.9	0.3	3.10E-36
CA00g92220	Hypothetical protein SORBIDRAFT_1138s002030	3.9	0.3	3.10E-36
CA00g81390	Hypothetical protein SORBIDRAFT_1138s002030	3.9	0.3	3.10E-36
CA00g92180	Hypothetical protein SORBIDRAFT_1138s002030	3.9	0.3	3.10E-36
CA00g81120	Hypothetical protein SORBIDRAFT_1138s002030	3.9	0.3	3.10E-36
CA00g94510	Hypothetical protein SORBIDRAFT_1138s002030	3.9	0.3	3.10E-36
CA00g91010	Hypothetical protein SORBIDRAFT_1138s002030	3.9	0.3	3.10E-36
CA00g92900	Hypothetical protein SORBIDRAFT_1138s002030	3.9	0.3	3.10E-36
CA03g27250	Betaine aldehyde dehydrogenase	2.5	0.2	9.50E-36
CA00g99220	Cytochrome P450 monooxygenase	4	0.3	1.70E-35
CA00g96010	Uncharacterized protein LOC104434852	4.4	0.3	3.30E-35
CA01g00900	RNA polymerase II transcriptional coactivator KELP	3.7	0.3	6.00E-35
CA03g21780	Zinc finger CCCH domain-containing protein 56-like	6.1	0.5	9.90E-35
CA05g03420	Histone H4	2.8	0.2	1.90E-34
CA03g34930	60s ribosomal protein l37-3	2	0.2	3.20E-34
CA10g00310	Calcium-binding protein PBP1-like	4.7	0.4	1.50E-33
CA07g18530	60s ribosomal protein l8-like	2.5	0.2	1.80E-33
CA00g68260	Ribulose-bisphosphate carboxylase oxygenase LSU	3.4	0.3	8.20E-33
CA04g00910	KIROLA-like	8.5	0.7	1.70E-32
CA02g20670	Uncharacterized protein At4g08330, chloroplastic-like	4.5	0.4	3.90E-32
CA10g04290	Hypothetical protein JCGZ_00471	4.4	0.4	8.50E-32
CA00g91020	Hypothetical protein AALP_AA2G024700	4.1	0.3	8.50E-32
CA00g91580	Hypothetical protein AALP_AA2G024700	4.1	0.3	8.50E-32
CA00g96040	Hypothetical protein AALP_AA2G024700	4.1	0.3	8.50E-32
CA00g70480	Probable fructose-bisphosphate aldolase chloroplastic-like	-4.4	0.2	1.10E-117
CA01g08070	Phosphoribulokinase, chloroplastic-like	-4.5	0.2	3.70E-83
CA10g04960	Chloroplast RUBISCO activase	-4.1	0.2	3.00E-66
CA10g02050	Chlorophyll a-b binding protein chloroplastic-like	-4.5	0.3	1.50E-65

Pepper ID	Gene Description	Log2 Fold Change	LFC SE	*P* adj.
CA02g23440	Ribulose -bisphosphate carboxylase oxygenase small subunit	-2.9	0.2	9.80E-64
CA03g29950	Chloroplast chlorophyll a-b binding protein	-4.1	0.2	9.70E-59
CA12g14230	ABC transporter F family member 5-like	-3.3	0.2	3.40E-58
CA02g21470	Ferredoxin-NADP leaf-type chloroplastic	-3.2	0.2	7.60E-56
CA10g22340	Magnesium-protoporphyrin ix monomethyl ester	-3.9	0.2	1.50E-55
CA07g16160	Peroxisomal-2-hydroxy-acid oxidase GLO1	-3	0.2	1.30E-54
CA03g29260	Calcium sensing chloroplastic	-4.4	0.3	1.10E-53
CA00g63920	Chlorophyll a-b binding protein chloroplastic-like	-3.7	0.2	3.90E-52
CA03g29890	Ribulose-phosphate-3-chloroplastic	-3.3	0.2	9.60E-52
CA05g20700	Chlorophyll a-b binding protein chloroplastic-like	-4.2	0.3	2.00E-51
CA00g58200	Chlorophyll a-b binding protein chloroplastic	-5.1	0.3	7.60E-51
CA03g24850	Indole-3-acetic acid-induced protein ARG2-like	-3.4	0.2	6.20E-49
CA01g06400	Uncharacterized protein LOC102591394	-3.3	0.2	1.30E-48
CA00g46800	Photosystem I reaction center subunit VI- chloroplastic	-3.7	0.3	1.00E-46
CA08g14370	Chloroplast ferredoxin	-3.1	0.2	1.80E-46
CA03g04480	Protein proton gradient regulation chloroplastic	-3.7	0.3	1.70E-45
CA01g06410	Uncharacterized protein LOC102591394	-3.2	0.2	1.50E-44
CA06g05750	Beta-carotene hydroxylase	-3.7	0.3	2.80E-44
CA02g24340	Serine mitochondrial	-2.6	0.2	5.90E-44
CA02g05510	Stress-induced protein 16	-3.1	0.2	1.80E-43
CA06g26270	Photosystem I reaction center subunit chloroplastic-like	-3.4	0.2	3.00E-43
CA07g15620	ADP-glucose pyrophosphorylase small subunit	-2.9	0.2	3.80E-43
CA01g17090	Chloroplast pigment-binding protein CP26	-3.2	0.2	7.80E-43
CA09g10320	Chlorophyll a-b binding protein chloroplastic-like	-3.3	0.2	1.00E-42
CA05g01250	Magnesium-chelatase subunit chloroplastic	-4.8	0.3	2.60E-41
CA07g10990	Chlorophyll a-b binding protein chloroplastic-like	-3.1	0.2	4.10E-41
CA01g03660	PGR5-like protein chloroplastic	-3	0.2	2.30E-40
CA04g22590	ND	-2.8	0.2	3.20E-40
CA07g13840	Protochlorophyllide chloroplastic-like	-2.8	0.2	3.90E-40
CA00g52220	Fructose-chloroplastic-like	-2.9	0.2	7.70E-40
CA01g20290	Carotene cleavage partial	-4.3	0.3	8.90E-40

Pepper ID	Gene Description	Log2 Fold Change	LFC SE	*P* adj.
CA00g47550	Chloroplast sedoheptulose-bisphosphatase	-2.5	0.2	2.10E-39
CA07g14520	ATP-dependent zinc metalloprotease FTSH chloroplastic	-2.8	0.2	7.00E-39
CA06g18800	Chlorophyll a-b binding protein chloroplastic-like	-6.2	0.5	7.20E-39
CA08g15590	Chlorophyll a-b binding protein CP24 chloroplastic-like	-4.5	0.3	2.20E-38
CA07g21500	Light inducible tissue-specific ST-LS1	-2.6	0.2	4.80E-38
CA02g10990	Zeaxanthin epoxidase	-5.1	0.4	1.50E-37
CA02g12050	Chlorophyll a-b binding protein chloroplastic-like	-4.4	0.3	5.40E-37
CA04g00760	Chlorophyll a-b binding protein chloroplastic-like	-3.3	0.3	6.60E-37
CA04g00770	Chlorophyll a-b binding protein chloroplastic-like	-3.3	0.3	6.60E-37
CA00g99380	Chlorophyll a-b binding protein chloroplastic-like	-3.3	0.3	6.60E-37
CA06g13880	ATP synthase subunit b chloroplastic-like	-2.4	0.2	1.00E-36
CA01g08110	Plastidal glycolate glycerate translocator chloroplastic	-3.4	0.3	1.50E-36
CA12g18670	Low quality protein: glycine dehydrogenase	-3.3	0.3	1.70E-36
CA00g15310	Linoleate 13s-lipoxygenase 2- chloroplastic	-4.5	0.3	1.70E-36
CA02g02740	Glyceraldehyde-3-phosphate dehydrogenase chloroplastic	-2.5	0.2	1.90E-36
