# Supplementary material for: Transcriptome analysis of two near-isogenic lines of bell pepper (Capsicum annuum) infected with bell pepper endornavirus and pepper mild mottle virus
Source: Front Genet. 2023 Apr 13;14:1182578. doi: 10.3389/fgene.2023.1182578 (PMC10133535; doi:10.3389/fgene.2023.1182578)
Supplement: Supplementary file 1 [file DataSheet1.zip › Supplementary_Table_4.pdf]

Supplementary Material

Table S4. One hundred highly differentially expressed genes that resulted from the transcriptome analysis of two bell pepper near-isogenic lines in BPEV-/PMMoV for condition BPEV-/PMMoV vs. BPEV-/Mock. Genes were selected based on the adjusted p value. BPEV+ = infected with bell pepper endornavirus, BPEV- = free of bell pepper endornavirus, PMMoV = pepper mild mottle virus, and ND = not determined. (Table continued).

Pepper ID	Gene Description	Log2 Fold Change	LFC SE	*P* adj.
CA04g18300	WRKY transcription factor 2	7	0.3	4.90E-109
CA00g61790	Protein ELF4-like 3-like	5.2	0.2	4.90E-109
CA03g06510	GDP-mannose 3 -epimerase	3.7	0.2	1.60E-91
CA00g83460	rRNA intron-encoded homing endonuclease	6.4	0.3	2.00E-72
CA00g91060	rRNA intron-encoded homing endonuclease	6.4	0.3	2.00E-72
CA00g81400	rRNA intron-encoded homing endonuclease	6.4	0.3	2.00E-72
CA00g93960	rRNA intron-encoded homing endonuclease	6.4	0.3	2.00E-72
CA00g91600	rRNA intron-encoded homing endonuclease	6.4	0.3	2.00E-72
CA12g18930	ANKYRIN repeat and protein kinase domain-containing protein 1-like	5.1	0.3	2.10E-65
CA08g13400	Uncharacterized protein LOC102600962	5.9	0.3	3.50E-65
CA00g83820	Tubulin alpha-3 chain	3.4	0.2	2.30E-61
CA03g23600	Hypothetical protein (mitochondrion)	5.9	0.4	5.30E-57
CA00g67540	Vesicle-associated protein 1-3-like	5.2	0.3	5.10E-56
CA03g36540	Pumilio homolog 5	4.4	0.3	2.50E-51
CA10g17080	Protein YLS9-like	3.3	0.2	3.00E-51
CA12g07360	Pentatricopeptide repeat-containing protein chloroplastic-like	3.9	0.3	1.60E-46
CA11g01730	Protein chloroplastic	3	0.2	1.70E-46
CA06g22700	CCR4 associated factor 1-related protein	3.9	0.3	1.90E-46
CA10g01770	Protein transport protein SEC61 subunit alpha-like	3.4	0.2	5.60E-45
CA06g26670	60s ribosomal protein l27-like	2.7	0.2	4.50E-43
CA03g27250	Betaine aldehyde dehydrogenase	2.8	0.2	6.10E-43
CA01g02010	PIN2 TERF1-interacting telomerase inhibitor 1 isoform X1	3.3	0.2	2.20E-42
CA00g95020	Hypothetical protein MTR_5g051150	4.3	0.3	1.80E-40

Pepper ID	Gene Description	Log2 Fold Change	LFC SE	*P* adj.
CA00g94500	Hypothetical protein MTR_5g051150	4.3	0.3	1.80E-40
CA06g11330	Aldehyde dehydrogenase	2.8	0.2	7.60E-40
CA00g16000	60s ribosomal protein l18A	2.7	0.2	1.00E-38
CA03g21780	Zinc finger CCCH domain-containing protein 56-like	6.4	0.5	3.20E-38
CA01g00900	RNA polymerase II transcriptional coactivator KELP	3.9	0.3	7.40E-38
CA03g21190	Protein transparent testa GLABRA 1	5.1	0.4	1.70E-37
CA06g07430	60s ribosomal protein l36-2-like	2.3	0.2	2.90E-37
CA08g03020	WRKY transcription factor	4.7	0.4	4.80E-37
CA07g18530	60s ribosomal protein l8-like	2.7	0.2	1.00E-36
CA01g22170	40s ribosomal protein s16-like	2.2	0.2	1.30E-36
CA10g00310	Calcium-binding protein PBP1-like	4.6	0.4	8.50E-33
CA00g96010	Uncharacterized protein LOC104434852	4.3	0.3	1.10E-32
CA04g17920	C2H2-type zinc finger protein	5.9	0.5	1.30E-32
CA03g20640	Hypothetical protein CICLE_v10027385mg, partial	4	0.3	1.10E-31
CA10g04290	Hypothetical protein JCGZ_00471	4.4	0.4	1.60E-31
CA00g99220	Cytochrome P450 monooxygenase	3.8	0.3	1.80E-31
CA02g20670	Uncharacterized protein At4g08330, chloroplastic-like	4.4	0.4	2.60E-31
CA08g17890	Uncharacterized protein LOC102591329	4	0.3	9.70E-31
CA00g16580	60s ribosomal protein l18A	2.5	0.2	1.10E-30
CA00g91590	Hypothetical protein SORBIDRAFT_1138s002030	3.6	0.3	1.40E-30
CA00g83490	Hypothetical protein SORBIDRAFT_1138s002030	3.6	0.3	1.40E-30
CA00g92220	Hypothetical protein SORBIDRAFT_1138s002030	3.6	0.3	1.40E-30
CA00g81390	Hypothetical protein SORBIDRAFT_1138s002030	3.6	0.3	1.40E-30
CA00g92180	Hypothetical protein SORBIDRAFT_1138s002030	3.6	0.3	1.40E-30
CA00g81120	Hypothetical protein SORBIDRAFT_1138s002030	3.6	0.3	1.40E-30
CA00g94510	Hypothetical protein SORBIDRAFT_1138s002030	3.6	0.3	1.40E-30
CA00g91010	Hypothetical protein SORBIDRAFT_1138s002030	3.6	0.3	1.40E-30
CA00g70480	Probable fructose-bisphosphate aldolase chloroplastic-like	-4.5	0.2	5.70E-121
CA01g08070	Phosphoribulokinase, chloroplastic-like	-4.3	0.2	4.40E-76
CA10g02050	Chlorophyll a-b binding protein chloroplastic-like	-4.5	0.3	2.10E-66
CA10g04960	Chloroplast RUBISCO activase	-3.9	0.2	1.80E-60

Pepper ID	Gene Description	Log2 Fold Change	LFC SE	*P* adj.
CA03g29950	Chloroplast chlorophyll a-b binding protein	-4.2	0.2	2.60E-60
CA10g22340	Magnesium-protoporphyrin IX monomethyl ester	-4	0.2	6.00E-60
CA02g23440	Ribulose-bisphosphate carboxylase oxygenase small subunit	-2.8	0.2	3.90E-57
CA12g14230	ABC transporter F family member 5-like	-3.1	0.2	8.90E-54
CA03g29260	Calcium sensing chloroplastic	-4.3	0.3	1.10E-51
CA03g04480	Protein proton gradient regulation chloroplastic	-3.9	0.3	2.60E-51
CA00g46800	Photosystem I reaction center subunit VI- chloroplastic	-3.9	0.3	4.10E-51
CA07g16160	Peroxisomal-2-hydroxy-acid oxidase GLO1	-2.9	0.2	2.10E-50
CA07g10990	Chlorophyll a-b binding protein chloroplastic-like	-3.4	0.2	9.10E-50
CA02g21470	Ferredoxin-NADP leaf-type chloroplastic	-3	0.2	1.80E-49
CA00g58200	Chlorophyll a-b binding protein chloroplastic	-5	0.3	3.20E-48
CA01g06400	Uncharacterized protein LOC102591394	-3.3	0.2	4.20E-48
CA03g29890	Ribulose-phosphate-3-chloroplastic	-3.1	0.2	9.50E-48
CA06g05750	Beta-carotene hydroxylase	-3.8	0.3	2.10E-46
CA06g26270	Photosystem I reaction center subunit chloroplastic-like	-3.5	0.2	4.20E-46
CA00g63920	Chlorophyll a-b binding protein chloroplastic-like	-3.4	0.2	5.70E-46
CA08g14370	Chloroplast ferredoxin	-3.1	0.2	5.80E-46
CA05g20700	Chlorophyll a-b binding protein chloroplastic-like	-4	0.3	8.80E-46
CA09g10320	Chlorophyll a-b binding protein chloroplastic-like	-3.4	0.2	2.20E-45
CA05g01250	Magnesium-chelatase subunit chloroplastic	-5.1	0.4	1.10E-44
CA07g15620	ADP-glucose pyrophosphorylase small subunit	-2.9	0.2	4.20E-44
CA01g17090	Chloroplast pigment-binding protein CP26	-3.3	0.2	3.20E-43
CA06g18800	Chlorophyll a-b binding protein chloroplastic-like	-6.6	0.5	6.10E-43
CA07g21500	Light inducible tissue-specific ST-LS1	-2.7	0.2	2.90E-42
CA01g06410	Uncharacterized protein LOC102591394	-3.1	0.2	1.60E-41
CA02g02740	Glyceraldehyde-3-phosphate dehydrogenase chloroplastic	-2.7	0.2	7.60E-41
CA08g15590	Chlorophyll a-b binding protein CP24 chloroplastic-like	-4.7	0.3	9.70E-41
CA07g13840	Protochlorophyllide chloroplastic-like	-2.8	0.2	3.80E-40
CA02g05510	Stress-induced protein 16	-2.9	0.2	2.80E-39
CA03g29990	Geranylgeranyl reductase	-4.5	0.3	4.60E-39
CA02g12050	Chlorophyll a-b binding protein chloroplastic-like	-4.5	0.3	2.70E-38

Pepper ID	Gene Description	Log2 Fold Change	LFC SE	*P* adj.
CA00g87430	Thioredoxin chloroplastic-like	-3	0.2	5.40E-38
CA02g24340	Serine mitochondrial	-2.4	0.2	6.00E-38
CA00g47550	Chloroplast sedoheptulose-bisphosphatase	-2.5	0.2	7.40E-38
CA06g13880	ATP synthase subunit b chloroplastic-like	-2.5	0.2	1.10E-37
CA02g10990	Zeaxanthin epoxidase	-5	0.4	3.10E-37
CA04g21580	Photosystem I reaction center subunit IV chloroplastic	-3.3	0.3	1.80E-36
CA02g26120	Chloroplast manganese stabilizing protein-II	-3.3	0.3	5.20E-36
CA10g01400	Chlorophyll a-b binding protein	-3.6	0.3	7.90E-36
CA04g22590	ND	-2.6	0.2	2.10E-35
CA00g52220	Fructose-chloroplastic-like	-2.7	0.2	2.40E-35
CA01g34780	Photosystem I reaction center subunit chloroplastic	-4	0.3	3.30E-35
CA07g14520	ATP-dependent zinc metalloprotease FTSH chloroplastic	-2.6	0.2	7.40E-35
CA02g07770	Oxygen-evolving enhancer protein chloroplastic	-2.4	0.2	7.90E-35
CA02g12070	Chlorophyll a-b binding protein chloroplastic-like	-5.5	0.4	9.90E-35
CA03g24850	Indole-3-acetic acid-induced protein ARG2-like	-2.8	0.2	5.10E-34
